# *How can i think of stroke when i don’t even have money to eat?*: barriers to primary stroke prevention among Nigerian suburban community-dwelling adults

**DOI:** 10.1186/s12889-026-26930-3

**Published:** 2026-03-09

**Authors:** Christopher Olusanjo Akosile, Ifeoma Uchenna Onwuakagba, Sochima Johnmark Obiekwe, Fatai Adesina Maruf, Chinedum Charles Onyenekwe, Nkechi Edit Chiejina, Chiebuka Emmanuel Okoye

**Affiliations:** 1 Department of Medical Rehabilitation, Nnamdi Azikiwe University, Awka, Anambra State Nigeria; 2 Department of Physiotherapy, Bowen University, Iwo, Osun State Nigeria; 3 Department of Physiotherapy and Sports Science, Kwame Nkrumah University of Science and Technology (KNUST), Kumasi, Ghana; 4 Department of Research, Medical Research Circle (MedReC), Bukavu, Sud Kivu Democratic Republic of Congo; 5 Department of Clinical Chemistry, Nnamdi Azikiwe University, Awka, Anambra State Nigeria; 6 Department of Nursing Sciences, Nnamdi Azikiwe University, Awka, Anambra State Nigeria

**Keywords:** Stroke, Primary prevention, Barriers, Community-dwelling adults, Nigeria

## Abstract

**Background:**

Stroke remains a leading cause of morbidity and mortality globally, with the greatest burden being borne by low- and middle-income countries such as Nigeria. Despite the high prevalence of modifiable risk factors, primary prevention strategies remain poorly implemented, and contextual barriers to prevention are underexplored.

**Objective:**

This study explored barriers to primary stroke prevention among high-risk adults in Nnewi, Anambra State, Southeastern Nigeria.

**Methods:**

A qualitative exploratory design was adopted, utilising in-depth interviews and focus group discussions among ten adults previously identified as at high risk of stroke in an earlier profiling study. Participants were recruited from Nnewi, a suburban industrial town in Anambra State, Nigeria. Data collection continued until thematic saturation was achieved. Transcripts were analysed using the General Inductive Approach to identify recurrent categories and themes.

**Results:**

Participants aged between 38 and 67 years, and were predominantly male with varied educational and occupational backgrounds. Seven themes emerged: low risk perception and poor screening, misconceptions about stroke risk, faith and informal cultural prevention practices, poverty and access barriers, work and time constraints, difficulty sustaining lifestyle change, and family and gender role influences. Most participants reported that their present health status reduced the need for further screening and believed that their past normal body test createda sense of security regardingn their health. Our participants also reported not deeming the risk screening as necessary, and prioritized other household needs over health screening. Stress and familiar responsibilities were reported as unequally distributed, and yet they were unwilling to eliminate some stressors due to cultural expectations.

**Conclusion:**

Stroke prevention in Nigeria is hindered by an interplay of misconceptions, cultural practices, poor health attitudes, and financial constraints. This study points to the urgent need for context-sensitive interventions that integrate cultural and religious considerations, improve community awareness, and address affordability barriers. Policy makers should strengthen primary health care, expand insurance coverage, and invest in culturally-tailored health education.

**Supplementary Information:**

The online version contains supplementary material available at 10.1186/s12889-026-26930-3.

## Introduction

Stroke remains a leading public health challenge worldwide. It is the second leading cause of death and the third leading cause of death and disability combined globally [1]. Despite advances in acute care and rehabilitation, stroke continues to account for approximately 11% of all deaths worldwide, causing about 5.7 million deaths annually in low- and middle-income countries (LMICs) [2, 3]. Between 1990 and 2021, the global burden of stroke rose sharply: new stroke cases increased by 70%, stroke deaths by 44%, people living with stroke by 86%, and disability-adjusted life years (DALYs) by 32%. Most of this burden falls on low- and lower-middle-income countries, which account for 87% of stroke deaths and 89% of DALYs [4], and the World Health Organization (WHO) has estimated that by 2030, 80% of all strokes will occur in LMICs, including Nigeria [5].

Stroke is a multifactorial disease, influenced by both modifiable and non-modifiable risk factors [6–8]. While advancing age, genetics, and sex are non-modifiable risks, about 90–98% of strokes are attributable to modifiable factors such as hypertension, dyslipidemia, diabetes mellitus, obesity, physical inactivity, stress, and unhealthy diets, etc. [5–8] which suggests the importance of a prevention strategy that targets individuals without prior stroke history through risk factor modification and population-based health interventions. Encouragingly, findings from high-income countries demonstrate that effective primary prevention strategies combined with public health measures can halve stroke incidence and mortality [9]. However, in LMICs, including many sub-Saharan African countries, such strategies remain poorly implemented, and findings reveal low levels of structured prevention programs, limited public awareness, and inadequate access to preventive health services [10–13].

Recognizing the global urgency, the United Nations in 2011 and the WHO Global NCD Action Plan (2013–2030) called on member states to prioritize non-communicable disease (NCD) prevention, including stroke, aiming for a 25% reduction in NCD-related mortality by 2025 [14]. Yet, most LMICs remain off track, reflecting insufficient adoption of sustainable, country-specific, and evidence-based prevention strategies [15]. Barriers such as a lack of time or training among healthcare providers, limited reimbursement for preventive services, and low risk awareness among communities have been reported [16] but beyond these systemic challenges, barriers at individual levels also impact stroke prevention. These challenges may be compounded in settings like Nigeria, where stroke prevention guidelines, often adapted from high-income contexts, may fail due to differences in culture, health infrastructure, and resource availability. Given the multifactorial and context-specific nature of these barriers, there is a need to explore them within local populations to tailor interventions effectively. This study, therefore seeks to explore the barriers to primary stroke prevention in Nigeria through a qualitative approach.

## Methodology

### Study design

This study adopted a qualitative exploratory design and utilized focus group discussions (FGDs) and individual interviews to capture participants’ views and experiences. Data were analyzed using the General Inductive Approach, which supports systematic coding and theme development aligned with the study objectives.

### Study location and setting

The research was conducted in Nnewi, a major suburban community in Anambra State, Southeastern Nigeria, renowned for its industrial activity and diverse population. Participants were drawn from a convenient sample of community-dwelling adults identified as being at high risk of stroke during an earlier stroke risk profiling study [17]. Recruitment was based on their prior classification as high-risk individuals. A total of ten participants took part in the study, including four participants in one focus group discussion and six participants in individual interviews. The sample size was guided by thematic saturation, with data collection continuing until no new themes or perspectives emerged. Guest et al. (2020) suggest that in relatively homogenous samples, saturation is often achieved within 6–12 interviews, supporting the adequacy of the present sample for exploratory qualitative inquiry [18].

### Data collection

Data were collected through both FGD and one-to-one interviews. FGDs were used to explore collective beliefs and enabled participants to build on one another’s responses to reveal commonly held attitudes and culturally embedded interpretations. The individual interviews provided a private space for participants to discuss personal experiences. Before participation, everyone provided written informed consent after a thorough explanation of the study’s objectives and procedures. All discussions and interviews were conducted primarily in English, which is widely spoken in the study setting. However, participants were allowed to provide additional clarification in Igbo where necessary to ensure that their views were accurately expressed and understood. In such instances, meanings were confirmed during the sessions to maintain consistency in interpretation.

The FGD session lasted approximately two hours and was conducted face-to-face, while individual interviews lasted about 45 min. All sessions were moderated by the lead author using the researcher-developed interview guide (refer to Appendix 1). Both the lead and second authors are physiotherapists with training and experience in health research and qualitative data collection. This professional background supported effective facilitation of discussions in a respectful and participant-led manner. The second author, who is proficient in spoken and written Igbo, documented contextual observations, including non-verbal cues and environmental influences, and supported clarification of participant responses where local language expressions were used.

The interview guide was reviewed by subject experts prior to data collection to ensure clarity, appropriateness, and alignment with the study objectives. Feedback from this review informed minor refinements before the commencement of the main study. Discussions and interviews were audio- and video-recorded with participants’ full awareness and consent. Permission for recording was explicitly included in the informed consent process, and participants were informed of their right to decline recording or withdraw at any stage without consequences.

### Data analysis

Data were analyzed using the General Inductive Approach, which facilitates the condensation of raw qualitative data into a concise format while ensuring strong alignment between the research objectives and emergent findings. Audio and video recordings were transcribed verbatim by the researcher, who assigned pseudonyms to all participants to maintain anonymity. Transcripts were coded, organised into categories, and subsequently developed into overarching themes. To ensure rigor, transcripts and recordings were reviewed by third and fourth persons before the codes and themes were refined through iterative discussions.

### Ethical considerations

The study adhered to ethical standards for research involving human participants. Written informed consent was obtained from all participants, including specific consent for audio and video recording. Confidentiality was maintained through anonymization during analysis and reporting, and all recordings and transcripts were securely stored with access limited to the research team.

## Results

**Fig. 1:**
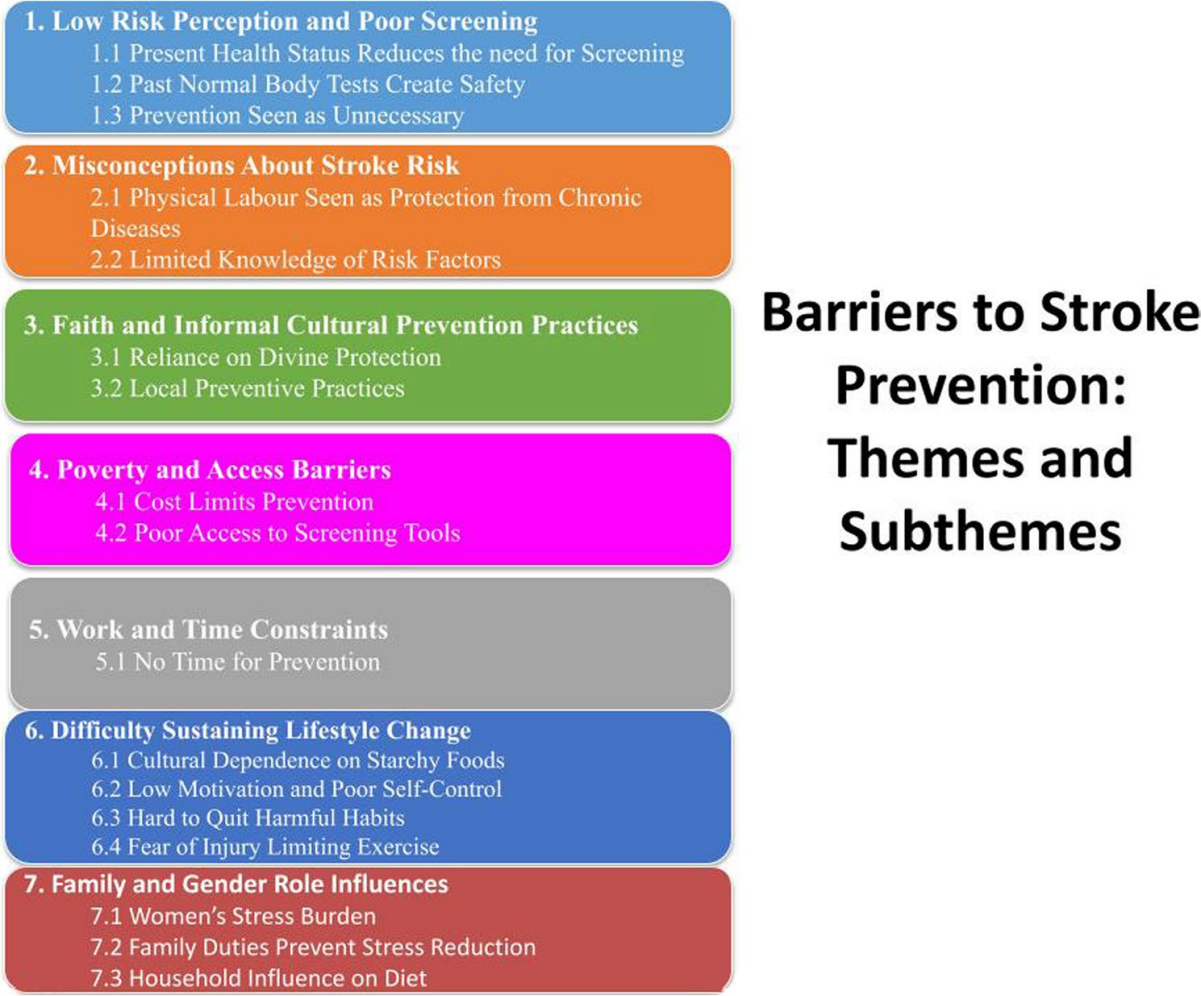
This figure enlists all the emerged themes and sub-themes

The socio-demographic profiles of participants are presented in Table 1. Participants were predominantly male and aged between 38 and 67 years, with varied educational and occupational backgrounds. The analysis of the data generated seven major themes and seventeen subthemes (Fig. 1). These themes reflect participants’ misconceptions about stroke prevention, socio-cultural beliefs, and structural barriers influencing readiness for lifestyle modification.

**Table 1:** Socio-demographic characteristics of participants in the focus group discussion

Serial Number	Highest Educational Attainment	Marital status	Occupation
1	Primary	Married	Trader
2	Secondary	Single	Public Servant
3	Secondary	Married	Trader
4	Primary	Married	Farmer
5	Tertiary	Married	Civil Servant
6	Tertiary	Married	Public Servant
7	None	Widowed	Trader
8	Primary	Married	Trader
9	None	Married	Trader
10	Tertiary	Married	Teacher

### Low risk perception and poor screening

Many participants expressed a low perceived susceptibility to stroke, which reduced their motivation to engage in primary prevention behaviors such as routine blood pressure or blood sugar monitoring. This theme was more evident among middle-aged and older adults (50–67 years), many of whom were traders, farmers, or public servants, whose everyday experiences may have contributed to how they interpreted health and illness. Our participants demonstrated a reactive rather than preventive orientation to health as they tended to view stroke prevention as unnecessary unless symptoms emerged.

#### Present health status reduces the need for screening

Many participants described themselves as being in good health and therefore saw little reason to check cardiovascular risk markers. This belief influenced their interest in routine screening for hypertension or diabetes and hospital visits, as they were often perceived as irrelevant in the absence of illness.

For instance, one middle-aged male trader explained:

*“I was told that I am not hypertensive… so I don’t check it again.”* (P2).

Similarly, other participants felt that the absence of previous heart-related conditions or diabetes meant there was no need for concern:

*“I haven’t seen the need… I haven’t had any heart-related conditions before.”* (P8).

*“I can’t check my blood sugar level because I don’t have diabetes.”* (P1)

Another participant, a male trader with primary education, also expressed a disengagement from formal healthcare services, indicating that hospital attendance was not considered necessary unless illness occurred:

*“I told you that I don’t visit the hospital; I don’t have any need to.”* (P1).

Even when screening had occurred, it was often infrequent and not prioritized, as reflected in one participant’s uncertainty about the timing of their last check:

*“I cannot recall the month… it was sometime this year.”* (P2).

#### Past normal body tests create safety

Rather than understanding stroke risk as dynamic and age-related, some of our participants interpreted past normal test results as long-term immunity or the elimination of risks.

One participant stated:

*“I was told that I wasn’t hypertensive…I was told that my body is free from or immune to it… If I am asked to check my BP whenever I am sick*,* I usually tell them that I was told that my body was free from hypertension”* (P2).

*“I can’t remember*,* but what I know is that I have checked for it before.”* (P3).

Such accounts suggest that some individuals still rely heavily on past encounters with health services without recognizing the need for ongoing monitoring, especially as stroke risk increases with age.

#### Prevention is seen as unnecessary

Our findings showed that engagement in preventive measures was strongly influenced by the value individuals attributed to them and their perception of relevance in aging, as well as commonly observed social attitudes toward health. A few participants explicitly described routine preventive checks as unimportant or not worth prioritizing.

For example, one participant stated:

*“I don’t see checking my heart status as an important exercise.”* (P2).

Others similarly emphasised that such practices were not considered necessary:

*“I don’t value it… I don’t deem it important.”* (P5).

*“…I don’t feel like it is important.”* (P8).

Notably, these views were expressed by participants with varying educational backgrounds (primary, secondary, and tertiary education) and different occupational statuses (trader, public servant, and civil servant), and suggest that low prioritisation of preventive monitoring may not be limited to a particular socio-economic or educational group, but may instead show cultural orientations toward reactive rather than preventive healthcare.

Another participant, who had no formal education, highlighted what was described as a “Nigerian factor,” where prevention is often delayed until symptoms become apparent:

*“You know the Nigerian factor*,* we don’t take things seriously unless it happens.”* (P7).

In addition, some participants questioned the relevance of lifestyle modification in later life. For instance, a 52-year-old participant expressed doubt about the importance of weight reduction at an older age:

*“At this age*,* should I still bother about weight?”* (P5).

### Misconceptions about stroke risk

Beyond low perceived susceptibility, participants’ accounts also revealed that misinformation and misconceptions about stroke risk influenced how they approached its prevention. Several participants drew on their everyday experiences, cultural interpretations of health, and incomplete health knowledge to explain why they believed certain behaviours were protective.

#### Physical labour seen as protection from chronic diseases

A few participants believed that strenuous physical work or persistent striving in daily life naturally protected them from conditions such as hypertension or diabetes. In this view, bodily exertion was not only part of livelihood but also seen as a form of cleansing or immunity against illness.

For example, a participant explained that his work as a painter provided health benefits through sweating, therefore considered it an automatic mechanism for preventing chronic disease, reducing the perceived need for routine medical monitoring.

*“My job as a painter involves working under the sun*,* which removes impurities… sugar is also removed from sweat…”*(P1).

Similarly, another participant expressed the belief that continuous stress and struggle could strengthen the body:

*“You get stronger and healthier when you continue to strive.”* (P4).

Such accounts indicate that some participants framed hardship and exertion as signs of resilience and health, rather than recognising that prolonged stress and uncontrolled cardiovascular risk factors may actually increase stroke vulnerability.

#### Limited knowledge of risk factors

Several participants also demonstrated gaps in knowledge regarding established stroke risk factors, particularly around cholesterol and cardiac health, which further influenced their perceptions of who is at risk and what preventive actions are necessary.

For instance, one participant associated high cholesterol only with body size:

*“It is fat people who have high cholesterol.”* (P1).

In addition, another participant reported that certain preventive practices, such as cardiac screening, were not even considered:

*“Such a thing (cardiac test) has not crossed my mind.”* (P2).

### Faith and informal cultural prevention practices

Rather than engaging in routine screening or evidence-based lifestyle interventions, a few participants indicated that faith-based and traditional health practices were sometimes perceived as sufficient for reducing stroke risk.

#### Reliance on divine protection

A few participants expressed strong religious confidence that serious health conditions, such as a stroke, would not happen to them. In these accounts, health outcomes were viewed as being under divine control, which reduced the perceived urgency of preventive action.

For instance, one participant stated:

*“God will not allow that to happen to me.”* (P1).

In addition, one participant explained that weight-related risks could be managed through deliberate abstinence:

*“I know. That’s why we should abstain from what can make us fat. We need to frequently fast….without waiting for the pastor to tell you to fast. You can just personally schedule fasting for yourself.”* (P2).

#### Local preventive practices

Alongside religious coping, several participants described informal practices, often based on local knowledge, personal experience, or community beliefs rather than medical advice, that they believed could prevent diabetes, overweight, or other stroke-related risks.

For example, one participant reported avoiding sugary drinks and consuming a common leaf believed to lower blood sugar:

*“I don’t take mineral drinks… I run away from sugary things…….I consume utazi (Gongronema latifolium)…”* (P3).

Fasting was again mentioned as a personal preventive strategy:

*“…We need to frequently fast…”* (P2).

Another participant described observing the use of lemon as a perceived weight-reduction method from her sister:

*“…she was taking lemon… she said it reduced weight…”* (P5).

### Poverty and access barriers

Poverty and limited access to nearby facilities were duly accounted for as a barrier in this study. The accounts of our participants indicated that the cost of screening, healthy food choices, and cardiovascular care frequently compete with household priorities. These barriers, even with the desire and willingness to engage in some of these preventive measures, added another layer to the failure in attaining them. Discussed in the following subthemes:

#### Cost limits prevention

Several participants reported that they can not afford the costs associated with the routine monitoring of these stroke risk factors. They often discussed these costs in relation to basic family responsibilities and suggested that preventive care was viewed as secondary to immediate needs.

*“I know that anything about the heart requires a very large amount of money.”* (P7).

One participant, a male trader aged 50 years, said;

*“…checking my cholesterol levels will be difficult for me…I haven’t got enough money to feed my children.”* (P1).

Similarly, another participant implied that screening would only be possible with external financial support:

*“Anybody that asks me to check my blood sugar will have to sponsor me.”* (P5).

*“Yes*,* I can go for a cardiac test… if the issue of money is settled.”* (P3).

Financial constraints also hindered the prioritization of dietary recommendations among our participants and even the ability to pursue weight reduction through healthier food options.

*“…Will you spend the family’s feeding money on large quantities of fruits for yourself alone?”* (P1).

*“Lack of money… if it’s fufu or garri that you have at home*,* you will join others and eat it because you don’t have an option.”* (P2).

*“How Can I Think of Stroke When I Don’t Even Have Money to Eat?*” (P9).

#### Poor access to screening tools

Beyond cost, participants described limited access to nearby health facilities and monitoring tools as another structural barrier to prevention. The effort required to leave work or travel to obtain screening discouraged routine checks, and this suggests that even when individuals may be willing to monitor their health, the limitations in proximity and unavailability of some of these devices hinder their success.

One participant, referencing his work setting, explained:

*“For me to leave my shop to go and look for where to check my sugar level… I don’t have the time to leave my shop.”*(P2).

Other participants also pointed to the absence of basic equipment as a key limitation:

*“…….Yes*,* having access to the equipment.”* (P9).

*“……availability of glucometers.”* (P10).

### Work and time constraints

For many participants, the challenge of preventing stroke was also influenced by their lives structured around demanding work routines, long hours, and responsibilities that leave little space for proactive health practices. In this context, prevention was often experienced as an “extra task” that competed with the immediate pressures of earning a living, meeting obligations, and coping with daily stress.

#### No time for prevention

Participants who were traders, teachers, and public servants identified time constraints as a direct barrier to routine health monitoring and physical activity.

One participant explained:

*“I can’t check my blood pressure because of my schedule.”* (P1).

*“I am always busy…”* (P10).

*“I don’t have time for exercise…”* (P2).

*“Yes*,* the problem is time…”* (P3).

Some participants described not eating fruits and late-night eating due to a lack of free time.

*“…Even if it’s 12 am that I return home and dinner is served*,* I will eat it.”* (P3).

*“Because*,* firstly*,* I am alone at home. So*,* I don’t have the time to fetch and prepare vegetables…”* (P6).

### Difficulty sustaining lifestyle change

Our participants’ accounts showed that even when stroke risk factors were recognised, making and sustaining healthy lifestyle changes was often experienced as difficult. Rather than describing lifestyle change as a simple personal choice, participants spoke about it as something influenced by cultural norms.

#### Cultural dependence on starchy foods

Some participants explained that reducing starchy foods was difficult because such meals are central to local dietary culture. Staple foods such as swallow were described not just as preferences, but as normal parts of everyday eating. These responses show a dietary pattern strongly influenced by cultural food practices and limited perceived substitutes.

One participant stated:

*“It will be difficult. You know that Igbos like swallow….starchy food*,* that’s what Igbos like.”* (P3).

Another participant raised a practical concern about what alternatives would even be available:

*“What will we be eating?”* (P2).

#### Low motivation and poor self-control

Our participants also described personal struggles with motivation and self-control, especially when tempted by desirable foods. Healthy intentions were often difficult to maintain in social or food-rich environments.

One participant vividly described this challenge:

*“Long throat. Once I see who is consuming meat*,* I would want to partake in it… once I see it*,* I can’t control myself… I will even eat more than others and beyond my capacity*,* especially if the food is free of charge.”* (P3).

#### Hard to quit harmful habits

On the accounts of two male public servants and traders aged 55 and 60 years respectively, they indicated that giving up certain harmful habits, such as high salt intake, would be difficult because these behaviours were strongly tied to personal taste and everyday practice. One also recognised that even when change was desired, it could not happen immediately and would require a gradual process over time.

*“I like salt to be tasted in my food. That’s where there is a problem… There must be salt in it.”* (P6).

*“It will be difficult. Gradually*,* I don’t think I can do them at once.”* (P7).

#### Fear of injury limiting exercise

In addition, exercise was perceived as unsafe by some participants who reported that fear of injury and previous negative experiences during exercise discouraged them from continuing with physical activity. These accounts also suggest limited familiarity with safe exercise practices and injury prevention strategies, which may further reduce confidence in engaging in regular physical activity as part of stroke prevention.

One participant explained:

*“There was a time I was engaging in exercise*,* but I injured my hands. I stopped since then.”* (P3).

Another participant similarly stated:

*“I left it after the injury.”* (P2).

### Family and gender role influences

From the accounts of our participants, we found that the gendered burden of stress emanating from family responsibilities and the household influence on behavior were also barriers that limit the primary prevention practices for stroke.

#### Women’s stress burden

Some of our participants suggested that stress exposure was not evenly distributed within households, with women perceived to carry a disproportionate burden. This gendered experience of chronic stress was described as a barrier to stroke prevention, as ongoing caregiving and domestic responsibilities may limit women’s ability to prioritise rest, stress reduction, or other preventive health behaviors.

A female participant emphasized that women experience excessive stress compared to men:

“This stress is more among women…This stress is excessive among the women.” (P3).

Similarly, a male participant highlighted how visible stress may affect women’s well-being over time:

“When you see such a woman, she would look 70, while the man, who usually is older than the wife, will look 30 because of the stress she is under.” (P 1).

These accounts suggest that chronic stress, particularly among women, may represent an important but difficult-to-address stroke risk factor within family life.

#### Family duties prevent stress reduction

Participants further described stress as tied to family and financial responsibilities, making stress reduction seem unrealistic. In this context, preventive recommendations such as “reducing stress” were not viewed as achievable because household survival and caregiving demands took priority.

A female participant explained that when she perceives stress could reduce:

“The stress cannot come down until after the children have grown up and started making their own money…thinking about house rent… will you lie down and sleep?” (P3).

A male participant linked stress to cultural expectations of masculinity and provision and suggested that reducing work-related strain could conflict with his role as a provider:

“If I am asked to reduce stress, it will burden my wife greatly. It is not easy. Any man being fed by a woman is an infidel. I can’t be an infidel. I must do something….you have to pay your children’s school fees.” (P 1).

#### Household influence on diet

Participants also indicated that preventive dietary change was also determined by household power dynamics rather than individual choice. For example, a female participant described how her husband’s preferences influenced the entire household:

“My husband likes sugar; consequently, he makes all of us eat it.” (P3).

## Discussion

One of the major barriers to stroke prevention identified in this study was poor knowledge of stroke prevention strategies and negative health attitudes and behaviors. Participants commonly expressed a false sense of security in their health and often equated past good health with current low risk. Some believed that only overweight individuals were vulnerable to cardiovascular risks such as high cholesterol, while others dismissed the need for regular health checks because of reasons such as time constraints, distance to health facilities, and unaffordable screening costs. In addition, unhealthy lifestyle behaviors, including poor dietary control, physical inactivity, and a lack of motivation to exercise, were also reported.

Our findings support several results in Nigeria as well as other global studies that indicate gross ignorance of stroke risk factors and prevention [19–24]. In North-Central Nigeria, for instance, Onah (2025) reported that awareness and recognition of stroke symptoms and risk factors ranged from only 20.6% to 47% of participants. Importantly, however, even higher levels of stroke awareness do not always translate into preventive practices [25]. Ogechi and Aina (2018) found that although 91.9% of respondents had heard of stroke, their understanding of modifiable risk factors was inadequate, and engagement in preventive behaviors remained poor [26]. Similarly, Arisegi et al. (2018) demonstrated that individuals with substantial knowledge still failed to practice effective prevention consistently, suggesting that knowledge alone is insufficient to change behavior [27].

To address these challenges, community-level interventions have been proposed. Participants in the study by Uvere et al. (2025) emphasized the importance of free risk factor screening and culturally tailored stroke awareness programs delivered through print, audio-visual, and electronic media [28]. This aligns with broader evidence that educational interventions significantly improve stroke prevention [29]. Nonetheless, limited access to mass media in underserved communities has been identified as a barrier to stroke awareness and, therefore highlighted that there could be structural issues beyond individual knowledge [26]. Addressing these inequities requires population-level campaigns that prioritize inclusivity, especially for individuals with lower levels of formal education, as stroke knowledge has been shown to strongly correlate with educational attainment [30].

Lack of time is a commonly reported barrier to healthy behaviors such as physical activity, and our findings support this [31–33]. However, in this study, time constraints extended beyond exercise alone. Participants described how work demands limited their ability to attend routine screening for stroke risk factors, engage in physical activity, and maintain healthy eating practices. Long working hours were often associated with delayed meals, late-night eating, and reliance on readily available foods rather than balanced diets. In the Nigerian context, some institutions have attempted to address access barriers through workplace-based clinics, such as those found in organizations like the Central Bank of Nigeria and the National Television Authority. These clinics bring basic healthcare services closer to workers and may reduce the time and effort required to seek care. Extending similar health services, such as basic screening and first aid, to large markets and other work settings may improve access to preventive care among informal workers who are often excluded from institutional health services. In addition, mobile clinics may offer a practical alternative for populations with limited time or mobility [34].

Poor perception and attitude towards stress handling emerged as another important theme in our study. Some participants expressed the belief that experiencing more stress was a sign of good health and that enduring high levels of stress was necessary to fulfill family responsibilities. They further described culturally rooted limitations in managing stress due to these responsibilities, with women often being unevenly burdened. To show that this was not a gender-biased observation, even a male participant acknowledged that stress and domestic responsibilities were more heavily distributed to women. This reflects the traditional Igbo family structure, where most household chores and caregiving roles are allocated to women, while men serve primarily as providers [35].

Another important factor we found was how some men associated reducing stress with diminished masculinity and abandoning their provider role. One participant reported that any man fed by a woman is an infidel. This finding aligns with Odimegwu et al. (2013), where participants described the social and physical costs of not conforming to traditional masculinity, including perceived mockery and loss of status [36]. The implication of this is the health cost, as some chronic conditions may be linked not only to poor health-seeking behavior but also to adherence to rigid traditional masculinity norms.

These findings highlight the importance of culturally responsive interventions aimed at addressing these misconceptions. It is also common in some families for men to send their wives to represent them at health sensitization programs. Therefore, it is imperative to deliberately target men within their own gatherings and social spaces. Odimegwu et al. (2013) also recommended that programs should include services that address men’s specific needs within their cultural context [36]. Most importantly, interventions should not focus on a particular age group alone, as traditional masculinity is transmitted across generations through parents, peers, schools, religious institutions, and media, and continues to shape health behaviors over time.

In addition, physically demanding jobs were often viewed as sufficient substitutes for deliberate health-promoting activities, including structured exercise. Despite these beliefs, participants also described feeling helpless in managing their work-related stress, which suggests a possible contradiction between perceived resilience and actual coping strategies. These findings also show a misunderstanding of the relationship between stress, physical activity, and health. Chronic and poorly managed stress has been consistently linked with adverse cardiovascular and psychological outcomes [37, 38]. Evidence suggests that both higher levels of reported stress and the perception that stress negatively impacts health are independently associated with worse physical and mental health outcomes. Importantly, the interaction between these two factors is harmful, as individuals who report high levels of stress and believe that stress strongly affects their health were found to have a 43% increased risk of premature mortality [37].

Also, the tendency to equate physically demanding work with health benefits also overlooks the differences between occupational physical activity (OPA) and leisure-time physical activity (LTPA). Evidence shows that OPA and LTPA affect health in very different ways. While OPA often increases the risk of long-term sickness absence and has been linked to poorer cardiovascular outcomes, LTPA consistently reduces these risks. For people living with cardiovascular disease, engaging in LTPA lowers the chance of recurrent events, type 2 diabetes, and even all-cause mortality. By contrast, high levels of OPA appear to worsen these outcomes. This misperception may partly explain why participants downplayed the importance of intentional stress management strategies and structured exercise [39–41].

These reported attitudes suggest that interventions must not only raise awareness of the negative health consequences of unmanaged stress but also promote adaptive coping mechanisms, including relaxation techniques, social support, and structured physical activity. Culturally sensitive educational programs that emphasize the distinction between harmful stress and beneficial physical activity may help correct these misconceptions and promote healthier perceptions of stress management.

Poverty was a recurring theme in the lived experiences of participants, emerging as one of the barriers to sustaining a healthy lifestyle. Many participants described how financial strain limited their ability to consistently purchase fruits and vegetables, which are central to maintaining cardiovascular health. In this context, poverty not only restricts dietary options but is compounded by the lack of proximity to secondary or tertiary care centers, affecting participants’ ability to consistently monitor cholesterol levels, blood sugar, cardiac health, and body weight. This finding is not surprising in the Nigerian context, where about 79.49% of older adults pay for healthcare services out of pocket [42]. For many, this reality may mean that scarce household income must be carefully stretched to cover immediate needs such as food staples, rent, or school fees for grandchildren, leaving little room for health-promoting choices.

The burden of out-of-pocket healthcare spending is quite concerning when viewed through the lens of chronic disease prevention. A study on the National Health Insurance Scheme (NHIS) in sub-Saharan Africa reported that such direct payments are a major reason behind uncontrolled hypertension, which in turn remains one of the most important risk factors for stroke. This implies that poverty among our participants may not only restrict food choices but also undermine medication adherence and access to preventive care and create a vicious cycle of risk.

Addressing these inequities calls for systemic and individual-level solutions. On the systemic level, urgent efforts should be made toward the effective implementation of Universal Health Coverage (UHC) through the NHIS to ensure that citizens can access standard healthcare services regardless of their income. At the individual and community levels, researchers and practitioners should recognize that interventions must be realistic and cost-sensitive. Campaigns promoting healthy lifestyles may have a limited impact if recommendations are financially unattainable for the population they intend to serve. Thus, designing cost-effective and culturally appropriate protocols should be prioritized. For instance, one strategy that may be practical could involve encouraging the consumption of seasonal fruits and vegetables, which are often more abundant and therefore cheaper at local markets. Such an approach may maximize the existing food systems and help families make healthier choices without overstretching their budgets.

From our findings, it appears that many participants believe stroke and cardiovascular disease prevention can only be achieved in tertiary hospitals. This shows a knowledge gap, as prevention largely depends on early detection and management of risk factors such as hypertension, diabetes, and obesity [43, 44]. Education and awareness programs should therefore emphasize how communities can maximize the use of their PHCs for regular blood pressure, glucose, and weight checks where such services are available.

Cultural and religious beliefs and practices strongly influence health perceptions and health-seeking behaviors, and our participants were no exception [45–48]. Several participants expressed the view that illness could be prevented or driven away through prayer and fasting. Dietary practices also followed cultural patterns. Among the Igbo tribe, which was the focus of our study, staple meals typically consist of swallow foods molded in lumps. These are predominantly carbohydrate-based, such as fufu (made from fermented cassava), ede (cocoyam), and nri ji (pounded yam), among others. Our participants commonly described these meals as the ‘real meals,’ while lighter foods such as fruits or vegetables were not considered sufficient. This perception raises concerns regarding the practice of balanced dieting among our participants. It may also help to explain findings from previous studies that report a high prevalence of overweight and obesity in this region [49–51].

A notable misconception among participants was the belief that being fat or thick-set is a marker of good nutrition. This view has been widely reported in Nigerian and other populations, where larger body size is often associated with wealth, strength, and well-being. However, from a biomedical perspective, this cultural ideal may increase vulnerability to conditions such as hypertension and diabetes. Traditionally, among the Pacific population, a larger body size was considered a sign of wealth, fitness, and good health [52–56]. More recently, a Kenyan study found that the participants reported that conclusions about a person’s health and wealth status are drawn based on different body sizes and traditional views about the ideal body size and societal pressure, as well as the women’s own experience with their body size, play a role in the perception of an ideal body [57]. Our finding supports that of Chigbu et al. in 2021, where they found nearly half, 44.07% of the population perceives large body size as desirable, and their positive perception of large body size significantly increases the odds of obesity by 1.5. Some 42.03% of obese persons even misperceived their weight to be normal, which further decreases the odds of weight-loss behavior [58]. These beliefs somewhat conflict with evidence linking obesity to increased risks of hypertension, diabetes, and cardiovascular diseases, hence calling for more targeted health education.

Although this study draws its strength from being the first study in Nigeria to explore the barriers to stroke prevention with a focus on primary stroke prevention, some limitations should be acknowledged. First, although the use of a qualitative exploratory design provided rich insights into participants’ perceptions, it limits the generalizability of the findings beyond the study setting. Second, participants were selected from a prior risk profiling study, meaning they were already classified as “high-risk,” which may have biased the findings toward more severe barriers compared to the general population. Third, the relatively small sample size with limited demographic spread, particularly the underrepresentation of women and the very elderly, may have restricted the diversity of perspectives captured. We also acknowledge the potential influence of social desirability bias given the context of this study and the researchers being healthcare professionals. Finally, as the study was conducted in Nnewi, a suburban and industrial hub with comparatively better access to healthcare, the barriers identified may differ from those experienced by older adults in rural or resource-poor communities, which may potentially limit the external validity of the findings. Future research should include larger, more diverse samples across both urban and rural settings, with balanced demographic representation, to provide findings that are more generalizable to the wider population of older adults.

## Conclusion

This study highlights that primary stroke prevention among high-risk adults in a suburban Nigerian setting is hindered by poor knowledge, entrenched cultural and religious beliefs, negative health attitudes, financial limitations, and limited access to screening services. Participants frequently underestimated their personal risk and prioritized immediate livelihood demands over preventive care. These findings show the need for culturally responsive and economically realistic interventions that directly address identified misconceptions while improving access to affordable risk screening at the primary healthcare level. Strengthening community-based health education and integrating stroke risk checks into routine primary care services may improve uptake. In addition, researchers should continue to generate locally grounded evidence and engage community and religious stakeholders in designing practical, low-cost strategies to reduce barriers to stroke prevention in Nigeria.

## Supplementary Information

Supplementary Material 1.

## Data Availability

The data that support the findings of this study are available from the corresponding author upon reasonable request.
