# Supplementary material for: How can i think of stroke when i don’t even have money to eat?: barriers to primary stroke prevention among Nigerian suburban community-dwelling adults
Source: BMC Public Health. 2026 Mar 9;26:1229. doi: 10.1186/s12889-026-26930-3 (PMC13085623; doi:10.1186/s12889-026-26930-3)
Supplement: Supplementary file 1 — Supplementary Material 1. [file 12889_2026_26930_MOESM1_ESM.docx]

**Interview guide**

1. What do you think causes strokes or increases someone’s chances of having it?
2. How would you describe your health, and what makes you feel that way?
3. Are there reasons why you check or don’t check for things like blood pressure, blood sugar, cholesterol, or heart health?
4. How do you feel about body weight and exercise; are they important for preventing sickness?
5. Are there any things you take or do that you believe help prevent illness?
6. What makes it difficult for you to check your health, eat better, or exercise regularly?
7. How does stress or your daily work affect your ability to take care of your health?
8. Are there certain foods in your culture that you feel are difficult to reduce or change? Why?
9. Are there healthy foods you know you should eat but find difficult to prepare or include in your meals?
